# Insights into the role of major bioactive dietary nutrients in lamb meat quality: a review

**DOI:** 10.1186/s40104-021-00665-0

**Published:** 2022-02-07

**Authors:** Javier Álvarez-Rodríguez, Olaia Urrutia, Sandra Lobón, Guillermo Ripoll, Juan Ramón Bertolín, Margalida Joy

**Affiliations:** 1 Departament de Ciència Animal, Universitat de Lleida, 25198 Lleida, Spain; 2 Departamento de Agronomía, Biotecnología y Alimentación, IS-FOOD Institute, Escuela Técnica Superior de Ingeniería Agronómica y Biociencias, Universidad Pública de Navarra, Pamplona, Spain; 3 Centro de Investigación y Tecnología Agroalimentaria de Aragón (CITA), Avda. Montañana 930, 50059 Zaragoza, Spain; 4 Instituto Agroalimentario de Aragón – IA2 (CITA-Universidad de Zaragoza), Avda. Miguel Servet, 177, 50013 Zaragoza, Spain

**Keywords:** Fatty acid, Forage, Oilseed, Polyphenol, Vitamin E

## Abstract

Feed supplementation with α-linolenic acid (ALA) and linoleic acid (LA) increases their content in muscle, ALA increases n-3 polyunsaturated fatty acids and decrease n-6/n-3 ratio in muscle, and LA increases rumenic acid. However, high LA supplementation may have negative effects on lambs’ lipid oxidative stability of meat. When the sources of ALA and LA are fed as fresh forage, the negative effects are counterbalanced by the presence of other bioactive compounds, as vitamin E (mainly α-tocopherol) and polyphenols, which delay the lipid oxidation in meat. There is a wide consensus on the capability of vitamin E delaying lipid oxidation on lamb meat, and its feed content should be adjusted to the length of supplementation. A high dietary inclusion of proanthocyanidins, phenolic compounds and terpenes reduce the lipid oxidation in muscle and may improve the shelf life of meat, probably as a result of a combined effect with dietary vitamin E. However, the recommended dietary inclusion levels depend on the polyphenol type and concentration and antioxidant capacity of the feedstuffs, which cannot be compared easily because no routine analytical grading methods are yet available. Unless phenolic compounds content in dietary ingredients/supplements for lambs are reported, no specific association with animal physiology responses may be established.

## Introduction

Bioactive compounds are substances which are present in foods in small amounts and have the ability to provide health benefits. Bioactive compounds include, but are not limited to, long-chain polyunsaturated fatty acids (PUFA), vitamins, carotenoids, peptides, and polyphenols, and their applications have been discussed in poultry and swine nutrition [1]. The interest in these dietary nutrients in sheep has increased over the last years, but it would be necessary to evaluate if these expected animal health benefits are also highlighted in meat quality.

Whilst the main sheep producers (China, Australia and New Zealand) raise heavy lambs yielding 17–23 kg carcass, some Mediterranean countries raise much smaller suckling and light lambs with 6–12 kg carcass [2]. The specific role of some bioactive nutrients in the feed sources on lamb meat quality may vary according to the feedstuffs and type of lamb production. This review aimed at evaluating the role of dietary unsaturated fatty acids (FA) and vitamins E, and polyphenols on lamb performance, color, FA profile and its pro- and anti- oxidant markers of meat.

## Unsaturated fatty acids

Ruminant meat is characterised by having a considerable percentage of saturated fatty acids, because PUFA from diet are subjected to a process of biohydrogenation conducted by ruminal microorganisms, thus the FA profile of the meat differs from that of in the diet. However, lamb meat presents low n-6/n-3 ratio compared with other meats such as pork and chicken (1.2 vs. 6.4 and 5.0, respectively) [3]. Also, it is an interesting source of conjugated linoleic acid (CLA), in particular the isomer *cis*-9, *trans*-11 (also called rumenic acid, RA), and α-linolenic acid (ALA, C18:3 n-3) and its elongation products. These FA have been related to the development of brain fetuses, the prevention of cardiovascular diseases and the reduction of carcinogenesis, atherosclerosis, the onset of diabetes and body fat mass [4, 5].

In the last decade, the use of dietary sources rich in PUFA, especially those of n-3 family, in lamb diets is of great interest, as a means to obtain a product more strongly fitted with the current human nutritional recommendations [6]. Various feeds, including oilseeds or plant and marine oils, have been investigated to improve the FA profile of lamb meat. These supplements are commonly rich in ALA (e.g. linseed, chia seed, camelina oil), linoleic acid (LA, C18:2 n-6) (e.g. corn oil, sunflower and safflower), eicosapentaenoic acid (EPA, C20:5 n-3) and docosahexaenoic acid (DHA, C22:6 n-3) (e.g. marine algae and fish oil) or oleic acid (OA, C18:1 *cis*-9) (e.g. canola/rapeseed and olive oil) [7].

Among ALA-rich sources, linseed (*Linum usitatissimum*) is one of the most studied feed ingredients. It contains ALA (around 56%), LA and OA (16% each) and saturated fatty acids (9.5%) [8, 9]. Chia seed (*Salvia hispanica* L.) is another novel feed that has been receiving attention because of its high ALA content. It includes up to 64% of ALA, LA (19%) and OA (9%) [8, 10]. Alternatively, the primary sources of EPA and DHA are marine oils derived from fish or algae. For livestock feeding, the most important microalgae species are *Arthrospira platensis, Chlorella* sp.*, Isochrysis* sp.*, Porphyridium* sp.*, and Schizochytrium* sp., the latter being mostly used [11, 12].

The inclusion of ALA-rich sources (linseed, chia seed and camelina oil) did not affect growth and carcass parameters, either in light [1, 2, 13, 14] or heavy lambs [15–17]. Similar results were observed when LA-rich sources (sunflower and safflower) were included between 2% and 15% in the diet [18–20], but the dry matter intake (DMI) tended to reduce with 15% of safflower inclusion [20]. In that sense, Oliveira et al. [21] observed that the inclusion of 30% of crushed sunflower caused a reduction of average daily gain (ADG) in heavy lambs, and worsen carcass characteristics. As pointed out by Nguyen et al. [9], high levels of fat in ruminant diets can result in negative effects on growth performance reducing DMI, due to a decrease in feed palatability, fiber digestibility and digestive nutrient flows. On the other hand, the results obtained about the inclusion of algae or fish oil are not conclusive. When algae (2% and 3.9%) or fish oil (3.3%) were included in the diet, studies showed a negative effect on lamb performance, with lower ADG and DMI and higher feed conversion ratio [1, 14, 22, 23] and lower carcass weights [14] than lambs fed the control diet. In contrast, there was no effect of inclusion of 1.9% [24], 3% [25] and 4% [14] of algae and 1.5% of fish oil [23] on lamb productive parameters.

The effects of dietary PUFA supplementation on meat quality characteristics of lambs are summarised in Table 1. The aim of supplementation of ALA-rich sources is to promote its content in *Longissimus thoracis et lumborum* (LTL) muscle of light and heavy lambs.

**Table 1:** The effect of different sources of polyunsaturated fatty acid (PUFA) supplementation on meat quality characteristics of *Longissimus dorsi* in lambs

Concentrate basal diet^2^	PUFA source^3^	Level, %	Fatty acid profile^4^						Oxidation, TBARS^**4**^	Sensory quality^**4**^					Ref.
			ALA	EPA	DHA	LA	OA	RA		Tend.	Juic.	Odor	Flavor	OL	
**Light lambs (< 24 kg body weight)**															
Barley/soybean	Ext. linseed	5	↑1.0	=	=	=	=	=	NR	NR	NR	NR	NR	NR	[1]
Barley/soybean	Ext. linseed	10	↑1.4	=	=	↓0.2	=	=	NR	NR	NR	NR	NR	NR	[1]
Barley/soybean	Ext. linseed	10.5	↑2.8	↑1.2	=	=	↓0.1	=	NR	NR	NR	NR	NR	NR	[1]
Barley/soybean	Chia seed	10	↑2.3	↑0.9	=	=	↓0.1	=	NR	NR	NR	NR	NR	NR	[1]
Barley/soybean	Ext. linseed	15	↑2.1	↑2.9	NR	=	=	NR	NR	NR	NR	NR	NR	NR	[2]
Barley/soybean	Ext. linseed	30	↑2.2	↑2.6	NR	=	=	NR	NR	NR	NR	NR	NR	NR	[2]
Barley/soybean	Ext. linseed	10	↑3.6	↑2.9	=	=	↓0.1	=	=	=	=	=	=	=	[13]
Barley/soybean	Ext. linseed+algae^5^	5 + 3.9	↑1.2	↑4.3	↑18.8	=	↓0.3	=	↑0.86	=	=	↓0.2	↓0.2	↓0.2	[13]
Barley/wheat	Ext. linseed	12.5	↑5.6	↑2.5	=	=	=	NR	=	=	=	↓0.2	↓0.1	=	[14]
Barley/wheat	Ext. linseed+algae	10.7 + 4	↑3.7	↑2.1	↑1.8	=	=	NR	↑2.1	=	=	↓0.2	↓0.1	=	[14]
Barley/wheat	Fish oil	3.3	=	↑9.6	↑11.0	↓0.3	=	NR	↑4.0	=	=	↓0.3	↓0.3	↓0.4	[14]
Barley/wheat	Algae^6^	2	=	↑6.6	↑12.4	=	↓0.1	=	NR	NR	NR	NR	NR	NR	[22]
Barley/soybean	Sunflower oil	4	↓0.3	=	=	=	=	=	NR	NR	NR	NR	NR	NR	[26]
**Heavy lambs (> 24 kg body weight)**															
Barley/corn	Ext. linseed	3	↑1.2	=	=	=	=	=	NR	NR	NR	NR	NR	NR	[27]
Wheat/triticale	Ext. linseed	3	↑1.0	=	=	=	↓0.1	=	NR	NR	NR	NR	NR	NR	[15]
Wheat/triticale	Ext. linseed	6	↑1.3	=	=	=	=	=	NR	NR	NR	NR	NR	NR	[15]
Wheat/triticale	Ext. linseed	9	↑2.5	=	=	=	↓0.1	=	NR	NR	NR	NR	NR	NR	[15]
Barley/soybean	Linseed oil	4.8	↑1.8	↑0.9	↑0.5	=	↓0.3	=	NR	NR	NR	NR	NR	NR	[16]
Barley/beet	Linseed oil	6	↑2.5	↑0.7	=	=	↓0.1	=	NR	NR	NR	NR	NR	NR	[17]
Barley/beet	Camelina oil	6	↑1.5	↑0.6	=	=	↓0.1	=	NR	NR	NR	NR	NR	NR	[17]
Barley/wheat	Fish oil	3	↑0.8	↑3.0	↑4.6	=	=	NR	NR	NR	NR	NR	NR	NR	[23]
Barley/wheat	Canola oil	3	↑1.4	↑0.4	=	=	↑0.2	NR	NR	NR	NR	NR	NR	NR	[23]
Barley/wheat	Fish oil+canola oil	1.5 + 1.5	↑0.9	↑2.8	↑2.5	↑0.7	=	NR	NR	NR	NR	NR	NR	NR	[23]
Commercial	Canola oil	150^7^	=	↑1.1	=	=	↑0.5	↑1.5	NR	NR	NR	NR	NR	NR	[28]
Soybean/barley	Canola oil+fish oil	2 + 1	NR	=	↑0.8	NR	NR	↑1.9	NR	↓0.3	=	=	=	↓0.2	[29]
Wheat/barley^8^	Fish oil	4.3	↓0.8	↑0.5	↑0.7	↓0.3	=	↓0.4	↑7.39	NR	=	NR	↓0.2	↓0.4	[30]
Wheat/barley^8^	Fish oil+algae^10^	2.1 + 1.6	↓0.8	↑1.3	↑3.7	↓0.2	↓0.2	↓0.5	↑9.09	NR	=	NR	↓0.3	↓0.2	[30]
Corn/wheat	Algae^5^	3	=	↑5.5	↑15.8	↓0.2	=	↑0.9	NR	NR	NR	NR	NR	NR	[25]
Oat/lupin grain	Algae^5^	1.95	=	↑0.5	↑4.9	=	↓0.2	NR	↑2.5	NR	NR	NR	NR	NR	[24]
Corn/soybean	Sunflower cake	10	=	=	NR	↑0.6	↓0.1	↑0.6	NR	↑0.1	↑0.2	NR	↑0.1	=	[21]
Corn/soybean	Sunflower cake	20	=	=	NR	↑0.6	↓0.1	↑0.6	NR	↑0.1	↑0.2	NR	↑0.2	=	[21]
Corn/soybean	Sunflower cake	30	=	=	NR	↑1.0	↓0.2	↑0.9	NR	↑0.2	↑0.2	NR	↑0.2	=	[21]
Barley/palm	Sunflower oil	2	NR	NR	NR	=	↑0.1	=	NR	NR	NR	NR	NR	NR	[18]
Barley/palm	Sunflower oil	4	NR	NR	NR	↑0.2	↑0.1	↑1.0	NR	NR	NR	NR	NR	NR	[18]
Corn/soybean	Safflower oil	7.5	=	=	=	=	=	=	NR	NR	NR	NR	NR	NR	[20]
Corn/soybean	Safflower oil	15	=	=	=	=	=	=	NR	NR	NR	NR	NR	NR	[20]
Corn/wheat	Safflower oil	3	=	=	=	=	↓0.2	↑0.6	NR	NR	NR	NR	NR	NR	[19]
Corn/wheat	Safflower oil	6	=	=	=	=	↓0.3	↑1.3	NR	NR	NR	NR	NR	NR	[19]
Barley/corn	Safflower oil	6	↓0.3	NR	NR	↑0.5	↓0.1	↑1.1	NR	NR	NR	NR	NR	NR	[31]

Effects are shown as relative variation with respect to the control diet and expressed in the fold change. *ALA* α-linolenic acid, *DHA* docosahexaenoic acid, *EPA* eicosapentaenoic acid, *Ext. linseed* extruded linseed, *IMF* intramuscular fat, *Juic*. Juiciness, *LA* linoleic acid, *NR* Not reported, *OA* oleic acid, *OL* overall liking, *RA* = rumenic acid; *TBARS* = thiobarbituric acid reactive substances; *Tend.* = Tenderness

^1^1-fold increase means 100% increase (it doubles)

^2^Main components of the concentrate are presented

^3^PUFA sources: ALA-rich = linseed, chia and camelina; LA-rich = sunflower and safflower; EPA/DHA-rich = algae and fish oil, OA-rich = canola

^4^Arrows indicate up (↑) or down (↓) significant values compared to control. Almost equal sign (=) symbolize no change between control and PUFA-rich diet

^5^DHA-Gold (Market Biosciences Corp., Columbia, MD, USA). Algal meal high in DHA derived from *Schizochytrium* spp. In Fan et al. [25] the algal meal is algae powder (*Schizochytrium* sp.; Xiamen Huison biotech Co., Ltd., Xiamen, China)

^6^TBARS were analysed in meat after 4 d display in Urrutia et al. [1] and Hopkins et al. [24] and after 7 d display in de la Fuente-Vázquez et al. [14] and Nute et al. [30]

^7^The inclusion level of canola oil was 50 mL/d, three times per week (150 mL/week)

^8^The effect of PUFA in this work is expressed as changes compared to the linseed oil supplemented group

^9^Lipid oxidation was assessed in *Semimembranosus* muscle

^10^Algae from a dinoflagellate of the class Dinophyceae (Martek Biosciences Corp., Columbia, MD, USA)

Overall, the results of the reviewed studies revealed that the inclusion of ALA-rich sources (linseed, chia seed and camelina oil) in the diet enhance ALA content in LTL muscle of light and heavy lambs. Consequently, total n-3 PUFA increased and n-6/n-3 ratio decreased significantly. Regarding EPA content in LTL muscle, findings are controversial, with some studies reporting no increase of EPA in lambs fed with ALA-rich sources and others reporting some signs of ALA elongation and desaturation, with increased EPA in light [1, 2, 14] and heavy lambs [16, 17]. However, most studies have reported that muscle levels of DHA did not increase, which could be the result of the limited conversion of ALA to their long chain products [29, 30]. Thus, the most effective strategy to enrich lamb meat in long chain PUFA, primarily DHA, may be the use of algae or fish oil in the diet [11]. Hopkins et al. [24] evaluated the inclusion of 1.95 g/kg of *Schizochytrium* sp. algae in LTL muscle of heavy lambs and observed an increase of 0.5-fold in EPA and 4.9-fold in DHA, which correspond to a value of 147.7 mg of the sum of EPA + DHA/100 g of meat.

Concerning OA content, some studies have reported a decrease in LTL muscle when ALA-rich sources are fed in light [1, 13, 22] and heavy lambs [15–17]. This may be attributed to the presence of high contents of dietary PUFA that inhibit the stearoyl-CoA desaturase (*SCD*) gene expression, as this gene encodes the enzyme responsible for the synthesis of OA from stearic acid (C18:0) and also RA derived from vaccenic acid (VA, C18:1 *trans*-11) [16, 32]. It has been suggested that dietary PUFA modulate the expression of genes involved in lipid metabolism, changing the adipocyte metabolism toward promoting dietary fatty acid uptake and activating the fatty acid oxidation, and downregulating de novo fatty acid synthesis acting on the expression of genes such as acetyl-CoA desaturase (*ACACA*), fatty acid synthase (*FASN*) or *SCD* [25, 32].

The enrichment of meat with PUFA that is beneficial from a point of human health view, affects the oxidative stability of meat, if the presence of some antioxidant compound, as vitamin E, is not adequate [33]. Lipid oxidation can influence key quality attributes, including color, texture, taste and aroma leading to rancidity and off-flavors, which ultimately affect meat acceptance [34]. There are not many studies examine the correlation between PUFA levels and meat lipid oxidation rates in lambs. Urrutia et al. [13] and de la Fuente-Vázquez et al. [14] reported that meat from light lambs supplemented with linseed (10% and 12.5%, respectively) did not exhibit lower oxidative stability in LTL muscle compared to controls, with concentrations of malondialdehyde (MDA) below 2 mg/kg of meat, which is reported to be the cut off value above which rancidity may be detected by consumers de la Fuente-Vázquez et al. [14]. In contrast, the partial substitution of linseed with algae (3.9 and 4% of algae, respectively) in the diet, significantly increased the lipid oxidation measured as Thiobarbituric Acid Reactive Substances (TBARS) levels. Similarly, Nute et al. [30] observed in heavy lambs fed algae produced the most lipid unstable meat on d 7 with a 9-fold increase in TBARS value, followed by that induced fish oil supplementation with 7.3-fold. The potential causes of the different effect of linseed and algae can be attributed to a high ALA content, which is susceptible to oxidation, but less than the EPA and DHA [35]. Also, it is known that plant oils as linseed contain antioxidants and increased α-tocopherol concentrations in muscle, preventing lipid oxidation [36].

The color of meat can be also affected by the high contents of some PUFAs. The inclusion of EPA and DHA-rich sources (fish oil and algae) in the diet, resulted in less lipid and color stability of meat during retail display [14, 30]. The latest authors reported that less red meat would be the consequence of increased metmyoglobin, caused by higher lipid oxidation due to higher n-3 long chain PUFA content in the diets. In contrast, linseed [2, 14, 27], canola oil [37], sunflower [21, 38] and safflower oil [19] dietary supplementation did not affect meat color parameters. Moreover, it has been found that redness increased when 15% of safflower oil [20] or 150 mL/week canola oil [28] were supplemented, which can be related to the increased intake of α-tocopherol from safflower oil and of monounsaturated fatty acids (MUFA) from canola fed animals, acting as limiting factor for lipid oxidation of meat.

The use of oilseed or plant and marine oils in the diet can influence sensory properties of meat [9]. Results from literature are not conclusive. The meat tenderness and juiciness were not affected by different PUFA-rich sources [13, 22, 30]. Despite this, Lima et al. [39] reported improved tenderness, juiciness and flavor when sunflower cake was included up to 30% in lambs’ diet. In contrast, 1% of fish oil addition was associated with low values for tenderness, resulting in the lowest overall sensory quality [29]. Generally, the use of fish oil and algae in the diet reduced the ratings for lamb odor, flavor and overall liking and increased fishy flavor, which was linked to the high proportion of EPA and DHA [13, 22, 30]. The inclusion of fish oil or marine algae up to 4% is the most effective strategy to improve n-3 long chain PUFA levels as EPA and DHA, however, it can negatively affect lamb performance and meat quality, with less appealing meat in terms of color, lipid oxidation and flavor.

In the last decades, the interest to reintroduce fresh forage in ruminant diets is increasing, since it is in line with the demands of consumers for animal products from natural, healthy and sustainable systems. Forages are naturally rich in ALA, and their intake can improve its deposition in lamb meat and therefore increase the PUFA content. There are many factors that can influence FA content of forages, such as the species, vegetation stage, conditions of conservation and N fertilization [40], with vegetation stage being the predominant factor.

Table 2 summarizes the amount of FA present in forages and consequently in lamb meat reported by several authors. The FA profile of forage influences the type of FA deposit on lamb meat. The natural pasture and legume forages present high amount of n-3 PUFA and, although they are subjected to ruminal biohydrogenation, partial amount of dietary unsaturated FA escape the rumen. Therefore, lambs fed with forages rich in n-3 PUFA present high content of these FA in meat and consequently the ratio n-6:n-3 is lower (Table 2). In contrast to other sources of PUFA, feeding forage does not increase lipid oxidation of meat (Table 2) as consequence of the presence of some bioactive compounds in the forages, as vitamin E (mainly α-tocopherol) and polyphenols, that act as natural antioxidants and can be transferred to the meat.

**Table 2:** Fatty acid composition of dietary forages and lamb meat (g FA/100 of total FA), and lipid oxidation (TBARS, mg MDA/kg of meat) of lamb meat

Treatment	Lamb type^4^	Fatty acid profile of forage				Fatty acid profile of meat			Oxidation, TBARS^5^	Ref.
		**C16:0**	**OA**	**LA**	**ALA**	**LA**	**n-3 PUFA**	**n-6:n-3**		
Mountain Pasture	Suckling	14.8	3.9	NR	51.9	2.5	7.0	2.0	1.20 (9 d)	[41]
Hay pasture	Suckling	16.5	9.0	NR	31.0	1.38	5.0	2.9	1.49 (9 d)	
Sainfoin (*Onobrichis vicifolia*)	Light	23.4	3.5	18.5	35.6	1.5	3.4	4.3	1.55 (14 d)	[42]
Alfalfa (*Medicago sativa*)	Light	24.7	3.5	16.3	40.8	1.4	2.4	3	1.96 (14 d)	
Total mixed ration^1^	Light	31.2	23.1	26.6	2.8	0.5	1.3	7	2.36 (14 d)	
Lowland^2^	Heavy	16.3	2.8	0.1	49.7	2.18	NR	1.88	NR	[43]
Alpine^3^	Heavy	15.5	3.7	0.1	48.6	3.21	NR	1.7	NR	
Alfalfa (*Medicago sativa*) silage	Heavy	18.5	2.7	19.6	36.2	2.9	1.3	NR	NR	[44]
Red colver (*Trifolium pratense*) silage	Heavy	15.1	3.4	18.1	40.2	3	1.1	NR	NR	
Birdsfoot refoil silage	Heavy	15.5	3.9	17.3	43.0	2.5	1.6	NR	NR	
Sainfoin (*Onobrichis vicifolia*) silage	Heavy	17.2	3.6	15.0	43.4	4.6	1.2	NR	NR	
Bladder cover (*Trifolium spumosum*)	Heavy	NR	NR	NR	12.0	3.8	8.7	1.8	3.1 (12 d)	[33]
Brassica (*Brassica napus*)	Heavy	NR	NR	NR	32.4	4.7	9.5	1.5	2.6 (12 d)	
Chicory (*Cichorium intybus*) + arrow leaf clover (*Trifolium vesiculosum*)	Heavy	NR	NR	NR	40.4	5.1	10.1	1.5	3 (12 d)	
Alfalfa (*Medicago sativa*) + Phalaris (*Phalaris aquatica*)	Heavy	NR	NR	NR	42.2	4.3	9.3	1.6	2.6 (12 d)	
Alfalfa (*Medicago sativa*)	Heavy	NR	NR	NR	29.4	4.6	9.5	1.5	3 (12 d)	

*ALA* α-linolenic acid, *FA* Fatty Acid, *LA* Linoleic Acid, *MDA* Malonaldehyde, *NR* Not Reported, *OA* Oleic Acid, *PUFA* Polyunsaturated Fatty Acid, *TBARS* Thiobarbituric Acid Reactive Substances

^1^50% barley straw, 11.6% corn, 11.5% barley, 9.3% legume, 7% rapeseed meal

^2^60% grasses, 40% legumes

^3^40% grasses, 20% legumes, 40% herbs

^4^Suckling is a 30–45 days old lamb with 12–14 kg, light is a 75–90 days old lamb with 22–28 kg, heavy is a > 120 days old lamb with > 35 kg of body-weight at slaughter

^5^TBARS values and days (d) of exposure are expressed

## Vitamin E

Vitamin E is synthesized only by plants, some algae and cyanobacteria [45], therefore, animals assimilate vitamin E from the diet. Vitamin E acts as a chain breaking antioxidant because is not degraded in the rumen, but it is accumulated within the lipid bilayers of cellular membrane increasing antioxidant potential [46]. There is a wide consensus that supra-nutritional vitamin E does not improve growth performance or carcass characteristics of light lambs [47–51], but its presence is a very useful way to delay the color fading, off-odors formation and lipid oxidation protecting PUFA and proteins from oxidation [48–52].

The magnitude of the effect of vitamin E depends on its concentration in muscle, which in turn depends on the content and chemical form of vitamin E ingested, the length of the feeding period and the kind of animal (light or heavy lamb). Since the 90’s it is known that supplementation of at least 1000 mg/kg with α-tocopherol to the lambs is effective in improving meat color and shelf-life stability [53], but every year new papers and reviews about supra-nutritional doses of vitamin E are still being published. Adjusting the concentration of vitamin E and time of feeding remains challenging because vitamin E is highly effective but expensive. The use of vitamin E to delay meat oxidation has many advantages over other antioxidants, vitamin E is mainly stored close to PUFA in the polar lipid fraction of muscle (phospholipids), the readiness to donate a hydrogen molecule to a free radical, the easiness of its use and supplementation, it is not degraded in the rumen, and finally, it is a compound which is not rejected by consumers. This section focused on supra-nutritional doses included in the feed, excluding intravenous dosage and industry by-products that may include vitamin E. The supra-nutritional doses are greater than 30 mg/kg of feed, the minimum concentration of vitamin E needed to prevent white muscle disease [54].

The most important question is: how many vitamin E in the muscle is needed to delay meat oxidation and spoilage? However, the answer depends on the variable studied. For colour stability, Jose et al. [55] reported that the threshold was 3.5 mg vitamin E/kg of meat. Redox activity was unaffected when the vitamin E, ranging from 2.5 to 5.1 mg/kg of meat, although the highest concentration delayed lipid oxidation [56, 57] and even with only 1.9 mg of vitamin E/kg of meat, Kasapidou et al. [58] observed a delay of lipid oxidation. The concentration of vitamin E in muscle required for the delay of the lipid oxidation varies between studies. López-Bote et al. [59] reported that the concentration of 5.4 mg of vitamin E/kg of meat was useful to control lipid oxidation, whereas Álvarez et al. [60] concluded that a concentration of α-tocopherol between 1.87 and 2.37 mg/kg meat, was effective to reduce lipid and pigment oxidation during long periods of conservation. Ponnampalam et al. [61] concluded that increasing lamb meat vitamin E content > 3.45 mg/kg muscle will delay oxidation across long term storage periods.

**Fig. 1:**
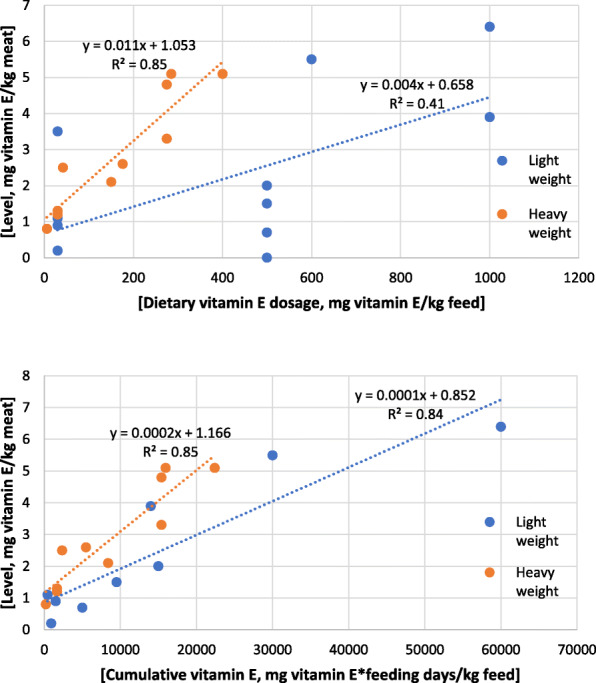
Relationship between doses of vitamin E (a) and days of feeding (b) with levels of vitamin E in meat [based on [56, 62–71, 125–127]]

It remains unclear how much vitamin E should be ingested to accumulate the desired vitamin E in muscle. Most of the cited studies provided the supra-nutritional doses and time of feeding but not the intake of lambs. The Fig. 1a shows the relationship between doses and concentration of vitamin E in muscle. There is no evident relationship between them, even considering the lamb body-weight. It can be supposed that heavy animals have more intramuscular fat and hence more vitamin E. However, Turner et al. [72] found a quadratic relationship between dietary vitamin E and muscle content, with a plateau around 5 mg of vitamin E/kg of meat, that was attained with a dietary vitamin E of approximately 400 mg/kg concentrate. Accordingly, Hampel et al. [52] carried out a meta-analysis of the relationship between tocopherol level in the diet and their effects on the quality of lamb meat. These authors reported that when dietary tocopherol levels were above 400 mg/kg of feed, there was no significant increase in the concentration of α-tocopherol in the meat. When both the time feeding lasted and dose supplemented is related to muscle content (Fig. 1b), the relationship is clearer although it could be either linear or quadratic, regardless of the slaughter weight of lambs.

Most of the studies have reported that supra-nutritional doses of vitamin E added to feedstuffs do not modify the proximal composition of meat but they indeed increase the vitamin E concentration in muscle. However, several studies on the effect of vitamin E against lipid oxidation reported effects, but it is difficult to compare them, due to the different doses and period of feeding vitamin E, as well as the vitamin E concentration in muscle and the different time of display/oxygenation of meat.

De Almeida et al. [62] reduced the meat oxidation from 0.12 to 0.08 mg MDA/kg meat without ageing or oxygenation but using a large feeding period (75 d) and great doses of vitamin E (1000 mg/kg of diet). González-Calvo et al. [63] feeding light lambs with 500 mg of vitamin E/kg of concentrate, observed that lipid oxidation of meat was delayed until 7 d of display when the concentration of vitamin E in meat was greater than 0.61 mg/kg of meat, but below this value, lipid oxidation increased exponentially. Ripoll et al. [64] found that feeding lambs with 500 mg of vitamin E/kg of concentrate for 33 d was enough to keep a value of TBARS lower than 0.2 mg MDA/kg of meat for 13 d of time of display. The threshold of concentration of vitamin E in muscle to delay the formation of metmyoglobin from 5 to 7 d was 0.91 mg vitamin E/kg meat [63]. With the same dosage of vitamin E but administered during 50 d, Ortuño et al. [65] decreased the oxidation from 4.6 to 0.4 mg MDA/kg of meat when time of display to oxygen was 7 d. The studies of Bellés et al. [66, 67] used 1000 mg vitamin E/kg of concentrate and reported lower values of MDA (mg/kg of meat) at 3 and 9 d of display in experimental treatments than control treatment. However, Leal et al. [68] only observed a significant effect of dosages of 250 mg vitamin E/kg feed but higher dosages did not improve lipid oxidation during 14 d of display. Baldi et al. [56] provided moderated dosages (285 mg vitamin E/kg of feed) during 56 d and observed lower values of lipid oxidation at 4 d of display (0.15 mg MDA/kg meat), mainly because the concentration of vitamin E in muscle was high (5.1 mg /kg meat) respect to the control. Finally, Simitzis et al. [69] fed lambs with a dose of 200 mg vitamin E/kg feed for 35 d and reported lower values of lipid oxidation than in control treatment on both fresh and frozen meat.

## Polyphenols

Plant polyphenols are secondary metabolites, which means they are metabolic intermediates or products no essential to plant life but rather required for interaction with the environment (i.e., produced in response to stress). They are classified according to their chemical structure and can be divided into four major classes: phenolic compounds, terpenes, sulphur and nitrogen containing secondary metabolites (as glycosides and alkaloids) [70]. We will focus on the first two groups due to their contrasting potential antioxidant effect on animal metabolism.

A phenolic is a chemical compound characterised by the presence of aromatic ring structure bearing one or more hydroxyl groups. Phenolics range from simple molecules, such as phenolic acid, to highly polymerised substances, such as tannins. Another way of categorising polyphenols is based on the chemical structures of their aglycones, that allow classifying them into four main groups: phenolic acids, flavonoids (isoflavones, isoflavonoids and chalcones, flavones, flavonols, flavanones and flavanonols, flavanols and proanthocyanidins, anthocyanidins), polyphenolic amides and miscellaneous group of non-flavonoid polyphenols (resveratrol, unique to the grapes and red wine, ellagic acid and its derivatives, found in berry fruits and in the skins of different tree nuts, and rosmarinic acid, a dimer of caffeic acid) [71].

### Proanthocyanidins

Proanthocyanidins (PACs) or condensed tannins (common name) are flavonoids formed by oligomers of flavan-3-ol units linked through carbon-carbon bound that resists the hydrolysis and they are the most common type of tannins found especially in the tree of the generous *Pinus* sp., *Acacia* sp., *Quercus* sp., in shrubs such as family of Rosaceae [73] and in forage legumes as sainfoin (*Onobrychis viciifolia*), sulla (*Hedysarum coronarium*) and birdsfoot trefoil (*Lotus pedunculatus*) [74]. They have been incorporated into the diet of sheep and lamb directly as fresh forage or indirectly as plant extracts of forages, for example, rockrose (*Cistus ladanifer*), or tree extracts in the concentrate, as quebracho (*Schinopsis balansae*).

The importance of PACs in ruminant nutrition is attributed to the fact that they can influence intake, production, health and product quality [75, 76]. In addition, they have a great capacity to interact with molecules, having adverse or beneficial effects on productive parameters, depending on their concentration, structure and molecular weight, as well as on the animal species and its physiology [73]. Besides of all these factors mentioned, there is a lack of standardization in the analysis, with special mention to the different standards used to quantify the amount of PAC. It is generally considered that the high content of PAC (> 50/kg dry matter) reduce the voluntary feed intake due to a reduction in palatability and digestibility, and consequently negatively affect productive parameters [74, 77].

In the last decades, PAC have been well-studied due to their recorded beneficial effects instead of their negative or anti-nutritive effects. The effect of PACs on growth and meat quality are presented in Table 3. The results related to the lambs’ growth are not conclusive. Studies showed an increase [84, 85], a decrease [86, 87], or no effect [88] on the ADG of lambs.

**Table 3:** The effect of the inclusion of different sources rich in proanthocyanidins in lamb’s diet on antioxidant activity, α-tocopherol and fatty acid profile in meat

Source	Dietary inclusion level	Lamb type^**1**^	Lambs growth^**2**^	Oxidation, TBARS^**2**^	α-tocopherol in meat^**2**^	FA profile^**2**^	Ref.
Quebracho (*Schinopsis balansae*)	100 g/kg DM	Suckling	=	↓ (9 d)	↑	↓ SFA	[41, 78]
Sainfoin (*Onobrichis vicifolia*)	Ad libitum (grazing)	Light	=	↓ (14 d)	↑	↑ CLA ↑ n-3 PUFA ↓ n-6:n-3	[42]
Rockrose (*Cistus ladanifer*)^3^	50, 100 and 200 g/kg DM	Heavy	=	↓ (7 d)	↑	↓ n-6 LC-PUFA ↓ n-3 LC-PUFA	[79, 80]
Mimosa (*Acacia mearnsii*) extract	40 g/kg DM	Heavy	=	= (7 d)	NR	=	[81]
Carob pulp (*Ceratonia siliqua*)	240 and 350 g/kg DM	Heavy	=	= (6 d)	NR	↑ n-3 PUFA ↓ n-6:n-3	[82]
Quebracho (*Schinopsis balansae*)	20 and 40 g/kg DM	Heavy	↑^4^	NR	NR	↓ SFA ↑ CLA ↑PUFA^5^	[18]
Sainfoin (*Onobrichis vicifolia*) silage	350 g/kg DM	Heavy	=	↓ (4 d)	↑	=	[83]

*CLA* Conjugated Linoleic Acid, *LC-PUFA* Long Chain Polyunsaturated Fatty Acids (sum of ≥ C20), *PUFA* Polyunsaturated Fatty Acid, *SFA* Saturated Fatty Acid, *TBARS* Thiobarbituric Acid Reactive Substances

^1^Suckling is a 30–45 days old lamb with 12–14 kg, light is a 75–90 days old lamb with 22–28 kg, heavy is a > 120 days old lamb with > 35 kg of body-weight at slaughter

^2^Arrows indicate up (↑) or down (↓) significant values compared to control. The almost equal sign (=) symbolize no change between control and proanthocyanidins-rich diet. TABRS values and days (d) of exposure are expressed

^3^Effects of the highest dose (200 g/kg DM) vs. 50 and 100 g/kg DM

^4^Only with an inclusion of 20 g/kg

^5^Only with an inclusion of 40 g/kg

Concerning the meat colour, the inclusion of 100 g/kg DM of quebracho in the concentrate of the dams diet (70:30 Forage:Concentrate) increased the lightness, yellowness and hue values in the meat of their suckling lamb compared to control on d 7 and 9 of air exposure [78]. Luciano et al. [89] also reported an increase in hue with an inclusion of 70 g/kg DM of quebracho in light lamb concentrate. However, this effect was not observed when the quebracho was included at 50 g/kg DM in the concentrate of light lambs [42]. In a recent meta-analysis, taking into account 41 original articles, Álvarez-Rodríguez et al. [90] concluded that the inclusion of PACs did not improve the colour of meat compared with the control.

Another desirable effect of the PACs is their antioxidant capacity [91], ascribed to their scavenging of free radicals, chelating of redox-active metals and inactivating of hemeproteins via reduction of highly pro-oxidant ferryl states [92]. López-Andrés et al. [93] reported that the PACs from quebracho were not absorbed in the gastrointestinal tract of light lambs, but induced antioxidant effects in their plasma and livers. The inclusion of rockrose at 50, 100, and 200 g/kg DM in the feed in fattening lambs diet did not affect the reducing ability (ferric reducing antioxidant power assay, FRAP) or the radical scavenging ability (trolox equivalent antioxidant capacity assay, TEAC) in muscle, however, the highest inclusion of rockrose showed lower levels of lipid oxidation (TBARS) compared to diets with lower inclusion levels. In this line, Lobón et al. [42] reported a considerable reduction of TBARS in light lambs whose dams grazed sainfoin than lambs whose dams grazed alfalfa or fed indoors a total mixed ratio during lactation, which may also be partly attributed to the increase in vitamin E content of muscle.

In addition, dietary PACs can modulate ruminal lipid metabolism by negatively affecting some species of fibrolytic bacteria and ciliate protozoa, and inhibit some steps of biohydrogenation of dietary PUFA [94]. Literature shows that the inclusion of PAC in the diet can be useful to improve the FA of meat, however the effect depends on the type of source and the amount intake (Table 3).

The inclusion of quebracho in the diet of heavy lambs decreased the total SFA and increased CLA [18] even when the diet was fed to dams and the effect was observed in suckling lambs [78]. An increase of n-3 PUFA content and consequently a decrease of n-6:n-3 ratio was observed in light lambs, that were in sainfoin meadows with their dams during lactation compared with lambs-dams in alfalfa meadows or indoors [42], and in heavy lambs with the inclusion of carob pulp (240 and 350 g/kg DM) in the concentrate [82]. In contrast, Jerónimo et al. [79] reported a decrease of n-6 and n-3 long chain PUFA with the inclusion of 200 g/kg DM of rockrose vs. 50 g/kg DM without affecting the total PUFA, while other studies reported no effect of FA in heavy lambs with the inclusion of 350 g/kg DM of sainfoin silage or 40 g/kg DM of mimosa [81, 83]. In a recent review, Frutos et al. [128] concluded that PACs can improve the concentration of certain potentially beneficial FA, such as ALA, LA, CLA, CLnA or VA, however they seem to induce smaller increases in other FA (e.g., C18:1 *t*10), that might be detrimental to animal performance (i.e., milk fat synthesis) and perhaps human health.

### Other phenolic compounds

Dietary inclusion of antioxidants in animal feed has been proven a more effective strategy to prevent oxidative damage of meat than their direct addition into meat [95]. There are several phenolic compounds extracts with a positive effect on meat stability that may be also considered as animal dietary supplements. Pomegranate pomace and tomato pomace extracts are used directly in the packing to increase the shelf life meat, but synthetic antioxidants (sodium ascorbate), grape and olive pomace aqueous extracts (dose of 1000 mg/kg of meat) have been recommended as natural additives in minced lamb meat patties, due to higher radical scavenging activity and lower metal chelating activity with respect to the first extracts mentioned [96]. Muiño et al. [97] concluded that the red wine extract was more effective as antioxidant in lamb meat patties than direct vitamin E addition, even when n-3 PUFA content was high. The antioxidant properties of red wine have been related to polyphenolic compounds, which include flavonoids, especially catechins and proanthocyanidins, but also non-flavonoids such as hydroxybenzoic acids, hydroxycinnamic acids, and stilbenes [98]. Generally, research works do not report short-chain phenolic compounds in dietary ingredients and supplements for lambs, which limits its specific association with animal physiology responses. Nowadays, it is accepted that, at least in humans, phenolic compounds can be either absorbed in the small intestine or secreted in bile, or reach the large intestine, where they are susceptible to modification by the microbial population, resulting in microbiota-derived metabolites with potentially beneficial health effects [99].

There is no clear effect of short-chain phenolic compounds on the meat lipid oxidation outcomes, depending on the feed supplements nature, its active molecules, inclusion level and storage conditions (Table 4). Besides, there is an important drawback about the determination of the antioxidant capacity. The wide heterogeneity of the analytical methods is partially responsible for the variability of results related to the inclusion of polyphenols in the diet. A diversity of tests has been developed, as the Folin–Ciocalteu assay, FRAP assay, the TEAC assay, the oxygen radical absorbing capacity (ORAC) assay, DPPH (2,2-diphenyl-1-picrylhydrazyl) radical-scavenging capacity assay, and ABTS (2,2′-azino-bis (3-ethylbenzothiazoline- 6-sulfonic acid) substrate peroxidase assay. In this regard, Ortuño et al. [106] found that FRAP and DPPH assays were more suitable than the TEAC assay for predicting meat oxidation and the resulting discolouration and rancidity.

**Table 4:** The inclusion of plant extracts or oils rich in miscellaneous polyphenols and lipid oxidation in lamb meat

Lamb type^1^	Supplement presentation	Feed supplement	Main attributed active molecules	Dietary inclusion level	TBARS differences compared with control^2^	Ref.
Suckling	Plant extract	Orange (*Citrus × sinensis*) and lemon (*Citrus × limon*) extract	Cyanidin 3-glucoside Cyanidin 3-(6-malonyl) glucoside Peonidin 3-(6-malonyl) glucoside	90 mg/kg of body-weight (mixed with water)	↓ (7-day refrigerated storage)	[100]
Light	Plant by-product	Red wine (*Vitis vinifera*) extract	Flavonoids Hydroxybenzoic and hydroxycinnamic acids Stilbenes (resveratrol)	900 mg/kg feed	NS (stored in MAP (70% O2/30% CO2) over 12 d at 4 °C)	[101]
Light	Plant by-product	Pomegranate (*Punica granatum*) by-product	Conjugated linolenic acids (CLnA) Ellagitannin (hydroxybenzoic acid) Flavonoids α-tocopherol	200 g/kg of feed	↓ (7-day refrigerated storage)	[102]
Heavy	Essential oil	Grape seed (*Vitis vinifera*)	Flavonoids (catechins, epicatechins, *trans*-resveratrol, and procyanidin B1) α-tocopherol Phytosterols	20 and 40 g/kg of feed	↓ (12-day refrigerated storage)	[103]
Heavy	Plant extract	Yerba mate (*Ilex paraguariensis*) extract	Caffeic acid, 5-caffeoylquinic acid, and rutin. Alkaloids and saponins.	40 g/kg of feed	NS (7-day refrigerated storage)	[104]
Heavy	Plant by-product	Pomegranate (*Punica granatum*) seed cake	Conjugated linolenic acids (CLnA) Ellagitannin (hydroxybenzoic acid) Punicalagin and gallic acid	115 to 235 g/kg of feed	NS up to 175 g/kg of feed but ↑ at 235 g/kg of feed (1-day storage)	[105]

*MAP* Modified-Atmosphere Package, *NS* Not Significant, *TBARS* Thiobarbituric Acid Reactive Substances

^1^Suckling is a 30–45 days old lamb with 12–14 kg, light is a 75–90 days old lamb with 22–28 kg, heavy is a > 120 days old lamb with > 35 kg of body-weight at slaughter

^2^Unless otherwise stated, refrigerated storage was conducted in polystyrene tray with oxygen-permeable polypropylene film at 4 °C. Any manuscripts detected differences in redness index (a*) of lamb meat due to feed supplement

In a recent study, Leal et al. [107] evaluated the effects of supplementation at the level of 5 g/kg of feed with either plant extracts from bay (*Laurus nobilis*), marjoram (*Origanum majorana*), oregano (*Origanum vulgare*), rosemary (*Rosmarinus officinalis*), thyme (*Thymus vulgaris*), turmeric (*Curcuma longa*), cumin (*Cuminum cyminum*), caraway (*Carum carvi*), dill (*Anethum graveolens*), cinnamon (*Cinnamomum zeylanicum*) and nutmeg (*Myristica fragans*) during 14 d in finishing lambs. These authors observed divergent results on the antioxidant status in the tissues (as assessed by the different methods), that may be either associated with a pro-oxidant effect of the plant extracts or with the specificity of the different assays. Cinnamon and rosemary plant extracts had the greatest polyphenol content but the antioxidant status of muscle was increased only with nutmeg dietary supplementation. In addition, these authors reported no effect of these plant extracts on growth performance of the lambs, although the length of supplementation period was too short (14 d).

In Mediterranean countries, a valuable source of bioactive phenolic compounds could be the olive (*Olea europaea*) leaves and cake extracts, that are rich in tyrosol and hydroxytyrosol [108] and oleuropein [109], as they have important antioxidant potential to scavenge free radicals and to avoid chain reactions. Under in vitro conditions, olive leaf was even better antioxidant properties than ascorbic acid on oxygen scavenging, at concentrations over 50 μg/mL of extract [110]. Another food supplement to enhance the shelf life of meat may be the stoned olive cake from oil pressing, that has been included up to 350 g/kg in feed to reduce lipid oxidation of light lamb meat [111], although the meat colour attributes were not evaluated in that study. In lamb meat patties, Muiño et al. [108] analysed the effect of adding an olive waste extract (100, 200 or 400 mg gallic acid equivalents/kg muscle) under high-oxygen modified atmosphere packs storage for up to 9 d at 4 °C. Addition of the olive waste extract delayed meat discolouration and lipid oxidation without affecting the overall liking score by consumers. However, most of these studies were conducted either with meat patties or did not evaluate voluntary feed intake and growth of lambs, thus, it is necessary to confirm whether these olive or wine co-products may affect animal performance. Olive cake and leafs, and red wine waste extracts may be a good source for the enrichment of the antioxidant characteristics of lamb meat and for the strengthening of circular economy in agroecosystems, although they require previous drying and processing to be used as feed supplements.

### Terpenes

Plant terpenes constitute a large class of natural products which are composed of isoprene units. Vasta et al. [112] detected sesquiterpene volatile compound markers (copaene and β-caryophyllene) in meat from heavy lambs supplemented with rosemary essential oil (*Rosmarinus officinalis*), rich in cineole, camphor and pinene, or desert wormwood essential oil (*Artemisia herba alba*), rich in chrysanthenone, camphor and thujone, at the inclusion level of 400 mg/kg of feed. In this regard, Serrano et al. [113] observed in beef that terpenes appeared to preferably accumulate in the perirenal and peritoneal fat, and that sesquiterpenes were retained more than monoterpenes or their oxygenated derivatives in the tissues of the calves receiving essential oils.

More recently, Moura et al. [114] demonstrated that dietary supplementation with copaiba (resin) oil (0.5 to 0.75 g/kg of feed), that is rich in sesquiterpenes as β-cariophilene, β-bisabolene, α-humulene, β-selinene, and α-bisabolol, can improve feed efficiency in feedlot heavy lambs with no effect on the meat colour. However, these authors did not evaluate its effect on lipid oxidation of meat. Realini et al. [115] studying the supplementation of heavy lambs with a mix of aromatic spices (lemon albedo, thyme, garlic) (inclusion level of 131 to 157 g/kg of feed) did not observe effect on the colour stability of meat but improved their liking scores by consumers, which suggests that plant extracts terpenes may affect the volatile organic compounds in meat, in agreement with the outcomes by Ortuño et al. [116] when adding rosemary diterpenes in light lamb feed.

Red meat cuts, such as lamb, are often packed in high-O_2_ protective atmospheres to maintain their redness for longer without considering the consequent enhancement of lipid oxidation. Rosemary diterpene extracts (rich in carnosic acid and carnosol) (inclusion level of 200 and 400 mg/kg of feed) contributed to the inhibition of volatile compound formation and rancidity of lamb meat stored under modified atmosphere package (MAP) (70% O_2_/30% CO_2_) during 14 d [116]. In a subsequent study, these authors found that rosemary diterpene extracts (inclusion level of 600 mg/kg of feed) had a lower deposition rate than vitamin E in lamb muscle and were completely degraded during cooking, thereby being less effective than dietary vitamin E in enhancing the oxidative stability of the yielded patties [65]. In another recent study, Leal et al. [68] demonstrated that, unlike vitamin E, neither dose nor protection of rosemary extract (embedded in a fat matrix) had an effect on production parameters, lipid oxidation or meat colour stability of lambs during the 14 d of storage under retail conditions. Accordingly, this may explain that dietary terpenes from rosemary essential oil failed to improve the rumenic acid content of meat [112, 117], that was indeed increased with dietary *Artemisia* essential oil supplementation [112] and grape seed oil supplementation [103]. As plant essential oils may contain not only terpenes but also some minor phenolics, these do not seem to modify rumen biohydrogenation to an extent enough to increase rumenic acid accretion, but they may decrease lipid oxidation in meat during long refrigerated storage (Table 5).

**Table 5:** Dietary inclusion of terpenes and lipid oxidation of lamb meat

Lamb type^1^	Supplement presentation	Feed supplement	Main attributed active molecules	Dietary inclusion level	TBARS differences compared with control^2^	Ref.
Suckling	Plant extract	Thyme (*Thymus zygis* ssp. *gracilis*) leaves	Thymol, carvacrol and eugenol	75 g of thyme leaves/kg of ewe feed	↓ (stored in MAP (70% O_2_/30% CO_2_) over 12 d at 4 °C)	[118]
Suckling	Plant extract	Carnosic acid from rosemary powder (470 g/kg purity)	Carnosic acid extract	0.096 g/kg of body weight (reconstituted in 25 mL/d of milk replacer)	↓ (14-day refrigerated storage)	[119]
Suckling	Plant extract	Astaxanthin-commercial powder	Astaxanthine	25 mg/kg of milk replacer	↓ (3-month frozen but not in 7-day refrigerated)	[120]
Light	Plant extract	Carnosic acid from rosemary powder (470 g/kg purity)	Carnosic acid extract	0.6 and 1.2 g carnosic acid/kg of feed	↓ (14-day refrigerated storage)	[119, 121]
Light	Plant extract	Mix of rosemary diterpenes	Carnosic acid and carnosol	200 and 400 mg/kg feed	↓ (stored in MAP (70% O_2_/30% CO_2_) over 14 d at 4 °C)	[106]
Light	Plant extract	Mix of rosemary diterpenes	Carnosic acid and carnosol	600 mg/kg feed	↓ (2-day refrigerated storage)	[65]
Light	Plant extract	Mix of rosemary diterpenes (with or without an hidrogenated fat embedded matrix)	Carnosic acid and carnosol Rosmarinic acid	200–800 mg/kg feed	NS (stored in MAP (70% O_2_/30% CO_2_) over 14 d at 4 °C)	[68]
Heavy	Essential oil	*Rosmarinus officinalis*	1,8 cineole Camphor Pinene (α- and β-)	400 mg/kg of feed	NS (7-day refrigerated storage)	[122]
Heavy	Essential oil	Artemisia herba alba	Chrysanthenone Camphor Thujone (α- and β-)	400 mg/kg of feed	NS (7-day refrigerated storage)	[122]
Heavy	Plant extract	Rosemary distillation residues	Diterpenes (carnosic acid and carnosol) Rosmarinic acid Flavonoids (Hesperidin)	600 and 870 g/kg of feed	↓ (14-day refrigerated storage)	[123]
Heavy	Essential oil	Mix of 1:1:1 cinnamon bark, eucalyptus leaves and dill seed	Monoterpene hydrocarbons Oxygenated monoterpenes Sesquiterpenes	3 ml in 300 g of concentrate	↓ (12-day refrigerated storage)	[124]

*MAP* Modified-Atmosphere Package, *NS* Not Significant, *TBARS* Thiobarbituric Acid Reactive Substances

^1^Suckling is a 30–45 days old lamb with 12–14 kg, light is a 75–90 days old lamb with 22–28 kg, heavy is a > 120 days old lamb with > 35 kg of body-weight at slaughter

^2^Unless otherwise stated, refrigerated storage was conducted in polystyrene tray with oxygen-permeable polypropylene film at 4 °C. Any of the manuscripts detected differences in redness index (a*) of lamb meat due to feed supplement, except Nieto et al. [118], who observed that redness index increased and Hue decreased in high dose thyme (75 g/kg of ewe feed) at 14 and 21 d of MAP storage (70% O_2_/30% CO_2_), and Morán et al. [121], who observed that Hue was lower with 1.2 g carnosic acid/kg of feed after 14 d of refrigerated storage

## Conclusions

Supplementation with dietary ALA-rich sources (accounting for 1–4% of ALA out of total diet composition) increases the content of ALA, n-3 PUFA and decrease n-6/n-3 ratio in muscle, without affecting animal performance, carcass and meat characteristics. Similar results are observed with LA-rich sources, that also increase LA and RA in meat, although high LA level may have negative effects on lambs’ performance and lipid oxidative stability. In contrast, in forage diets (especially fresh) the high dietary ALA and LA content (1–2% of total diet composition) is probably traded-off by the presence of other bioactive compounds, as vitamin E (mainly α-tocopherol) and polyphenols, that counterbalance the lipid oxidation in meat.

There is a wide consensus on the capability of vitamin E delaying lipid oxidation on lamb meat. Also, it seems there is a plateau of 4 to 5 mg of vitamin E/kg of meat, that can be reached with dietary doses of 400 mg of vitamin E/kg of feed. It is mandatory to adjust the vitamin E level in the concentrate and time of feeding to attain optimal concentration of vitamin E in muscle, but many studies do not provide the concentrate intake by lambs.

A high dietary inclusion of proanthocyanidins, phenolic compounds and terpenes reduce the lipid oxidation in muscle and they may improve the shelf life of lamb meat, probably as a result of a combined effect with dietary vitamin E. However, the recommended dietary inclusion levels depend on the polyphenol concentration and antioxidant capacity of the feedstuffs, that cannot be compared easily because no routine analytical grading methods are yet available. Unless phenolic compounds content in dietary ingredients/supplements for lambs are reported, no specific association with animal physiology responses may be established.

## Data Availability

The datasets used and/or analysed during the current study are available from the corresponding author on reasonable request.

## Abbreviations

ADG: Average daily gain
ALA: α-Linolenic acid
CLA: Conjugated linoleic acid
DHA: Docosahexaenoic acid
DMI: Dry matter intake
EPA: Eicosapentaenoic acid
Ext. Linseed: Extruded linseed
FA: Fatty acid
IMF: Intramuscular fat
Juic.: Juiciness
LA: Linoleic acid
LC-PUFA: Long chain polyunsaturated fatty acids (sum of ≥ C20)
LTL: *Longissimus Thoracis Et Lumborum*
MDA: Malonaldehyde
MAP: Modified-atmosphere package
NR: Not reported
NS: Not significant
OA: Oleic acid
OL: Overall liking
PACs: Proanthocyanidins
PUFA: Polyunsaturated fatty acid
SFA: Saturated fatty acid
RA: Rumenic acid
TBARS: Thiobarbituric acid reactive substances
Tend: Tenderness
